# Transparent and flexible fingerprint sensor array with multiplexed detection of tactile pressure and skin temperature

**DOI:** 10.1038/s41467-018-04906-1

**Published:** 2018-07-03

**Authors:** Byeong Wan An, Sanghyun Heo, Sangyoon Ji, Franklin Bien, Jang-Ung Park

**Affiliations:** 1 School of Materials Science and Engineering, Samsung Display-UNIST Center, Wearable Electronics Research Group, Ulsan National Institute of Science and Technology (UNIST), Ulsan Metropolitan City, 689-798 Republic of Korea; 2 School of Electrical Engineering, Samsung Display-UNIST Center, Ulsan National Institute of Science and Technology (UNIST), Ulsan Metropolitan City, 689-798 Republic of Korea

## Abstract

We developed a transparent and flexible, capacitive fingerprint sensor array with multiplexed, simultaneous detection of tactile pressure and finger skin temperature for mobile smart devices. In our approach, networks of hybrid nanostructures using ultra-long metal nanofibers and finer nanowires were formed as transparent, flexible electrodes of a multifunctional sensor array. These sensors exhibited excellent optoelectronic properties and outstanding reliability against mechanical bending. This fingerprint sensor array has a high resolution with good transparency. This sensor offers a capacitance variation ~17 times better than the variation for the same sensor pattern using conventional ITO electrodes. This sensor with the hybrid electrode also operates at high frequencies with negligible degradation in its performance against various noise signals from mobile devices. Furthermore, this fingerprint sensor array can be integrated with all transparent forms of tactile pressure sensors and skin temperature sensors, to enable the detection of a finger pressing on the display.

## Abstract (web summary)

Next-generation mobile security devices require fingerprint sensors that can be incorporated directly into the display. Here, Park et al. demonstrate a highly transparent, multifunctional capacitive fingerprint sensor array that simultaneously detects tactile pressure and finger skin temperature.

## Introduction

As mobile devices such as smartphones and smart watches become more ubiquitous and utilized in diverse areas of our daily lives, the importance of personal security on these devices is also rapidly escalating. Biometrics, which uses information about the human body, can be used to provide security on these smart devices because of the unique inherent characteristics of every person. Biometrics usually refers to technologies for measuring and analyzing the characteristics of the human body, such as retinas, irises, voice patterns, facial patterns, and fingerprints, which are unique for each person. Therefore, biometrics is a promising approach for ensuring user privacy. In terms of technical difficulties and cost issues, fingerprint recognition is the preferred technique among those that have been implemented so far. A variety of physical mechanisms have been exploited to capture electronic images of a human fingerprint, including optical, capacitive, pressure, and acoustic methods^1^. Optical fingerprint sensors use frustrated refraction over a glass prism. The finger is illuminated from a light-emitting diode (LED), while a photodetector transmits the image through a lens^2,3^. Thermal-detection-based fingerprint sensors can be made from a pyroelectric material that can detect temperature differences. This sensor scans the surface of the finger, measuring the heat transferred from the sensor to the fingerprint^4,5^. In the case of pressure-type fingerprint sensor, the principle of sensing is based on the piezoelectric effect. When a finger is placed over the dielectric top surface of the sensor, only the ridges come in contact with the individual sensor cells^6^. Ultrasound fingerprint sensors use the principle of medical ultrasonography in order to create visual images of the fingerprint. Ultrasonic sensors use high-frequency sound waves to penetrate the epidermal layer of skin. The reflected ultrasonic energy is measured using piezoelectric materials^7,8^. The capacitance is changed due to the distance of each ridge (closer) or valley (further) from the fingerprint sensor. Thus, a fingerprint image can be determined by the measurement of this voltage output signals over time at each capacitor of the sensor array^9^. Currently, capacitive fingerprint sensors that are used in mobile devices, especially in smartphones, are still opaque, and implemented either within an activation button or in areas on the back of these devices. In order to enhance the usability of mobile devices, a display that occupies a relatively larger area of the total device size has the highest priority in terms of product design. Thus, apart from the display, the space needed for other components (e.g., bezels, buttons, and sensors) needs to be reduced or completely eliminated on the front side of products^10^. As such, the development of transparent fingerprint sensors within a display is highly sought after. These invisible sensors can allow users to simply place their finger on the screen and identify the print, rather than on a button. For the transparent form of capacitive fingerprint sensors, transparent electrodes with high electrical conductance and high optical transmittance (*T*) are essential and necessary for sensor operations in high-frequency ranges. The high operation frequency (~1 MHz) of the fingerprint sensor can distinguish noise from the display (<200 kHz)^11^. Also, the high conductance of the transparent electrode can minimize the delay between the two adjacent electrodes. However, the sheet resistance (*R*_*s*_) of conventional transparent electrode materials, such as indium tin oxide (ITO), carbon nanotubes, graphene, fine metal meshes, or metal nanowires, is too high to allow for high-frequency signals that drive the capacitive fingerprint sensors in terms of noise from mobile devices. Moreover, in the case of metal electrodes, the width of electrode lines is limited in terms of obtaining high transparency due to their nontransparency. As a result, the capacitance change between fingerprint ridges and valleys is very low.

Here, we report an unconventional approach for the fabrication of a transparent, flexible fingerprint sensor array with multiplexed detection of tactile pressure and finger skin temperature for mobile devices. Transparent, flexible electrodes of this multifunctional sensor array were formed using random networks of a hybrid nanostructure based on ultra-long silver nanofibers (AgNFs) and fine silver nanowires (AgNWs)^12–14^. These invisible percolative networks exhibit excellent optoelectronic properties (*R*_*s*_ of ~1.03 Ω/sq *T* of 91.04% in the visible light region) and outstanding reliability against mechanical bending. In addition, the fingerprint sensor using the AgNF–AgNW hybrid electrode has high resolution (318 capacitors per inch (CPI)) with good transparency (89.05%). This resolution sufficiently satisfies the criteria set by the Federal Bureau of Investigation (FBI) for extracting fingerprint patterns (resolution > 250 CPI)^15^. The individual single cell of this sensor array (covered by a top passivation layer with a thickness of 100 μm) presents static capacitance of 100 ± 0.05 fF under an untouch condition, and detects 4.2 ± 0.07 fF of the capacitance variation between the ridge and valley of a fingerprint under a touch condition. Also, our sensor array operates reliably at a high frequency (1 MHz) with negligible degradation in its performance against noise signals from mobile devices. In order to prevent the fingerprint forgery using artificial fingerprints, temperature of human finger skin can be detected using temperature sensors to distinguish real and counterfeit fingerprints with improving security levels further. In addition, to replace the operation of pressing the activation button of smartphones with a finger, transparent pressure sensors were located on the display for sensing tactile pressures. For this purpose, pressure-sensitive field-effect transistors (FETs) were formed using the transparent layers of the oxide–semiconductor channel and the dielectric elastomer with the transparent AgNF–AgNW electrodes and located between the transparent fingerprint sensor array. Pressure-sensitive FETs were formed using the transparent layers of the oxide–semiconductor channel and the dielectric elastomer with the transparent AgNF–AgNW electrodes, and the thickness of this elastomer was decreased by applying pressure with the increasing capacitance of the metal–elastomer–semiconductor structure. In addition, a transparent temperature sensor was also integrated into this array to monitor the temperature range of human finger skin, which enables the recognition of artificial fingerprints, thus improving security.

## Results

### Fabrication process of the multiplexed fingerprint sensor

**Fig. 1:**
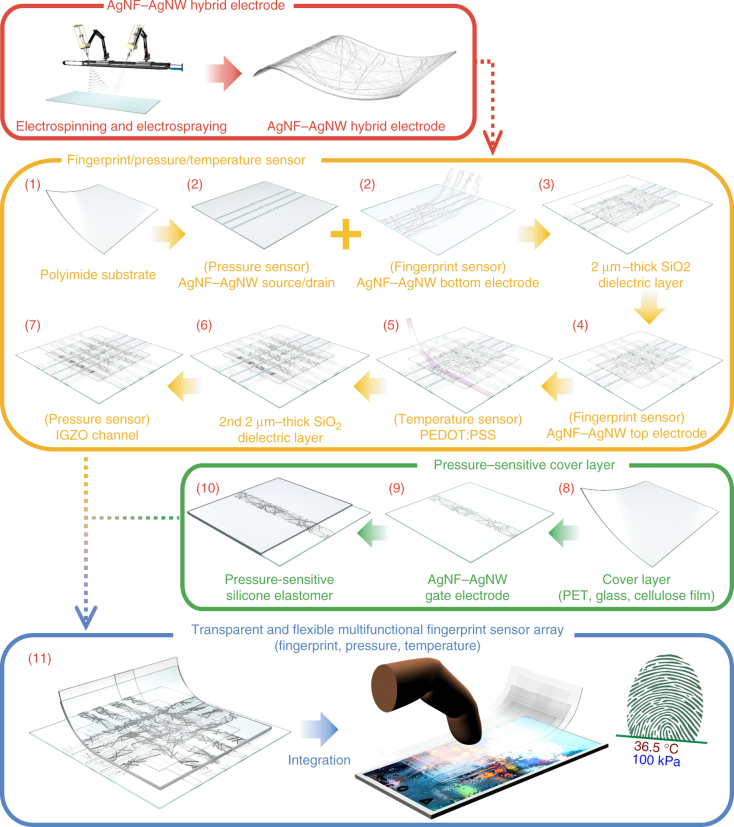
The fabrication process for the multiplexed fingerprint sensor

Figure 1 and Supplementary Figs. 1–3 show the overall fabrication process for this multifunctional fingerprint sensor array. The red numbers in Fig. 1 were related to Supplementary Figs. 1–3 to increase the clarity of Fig. 1. In the first step of the fabrication, a suspension of Ag nanoparticles (NPK Korea, average diameter: 40 ± 5 nm, solvent: ethylene glycol, concentration: 50 wt%) was electrospun continuously onto a colorless polyimide (c-PI) film (thickness: 25 μm) using a nozzle (inner nozzle size: 0.33 mm, outer nozzle size: 0.64 mm), and then thermally annealed at 150 °C for 30 min to coalesce the Ag nanoparticles into electrically conductive AgNFs with an average diameter of 338 ± 35 nm (Supplementary methods)^12, 13, 16^. This thermal annealing step did not break the AgNFs, and the single fibers were long enough to minimize the number of junctions between one-dimensional metallic geometries, which leads to a significant reduction of *R*_*s*_ while maintaining large open spaces in the networks for high transmittance. However, these large open areas can significantly increase the resistance of AgNF networks when they are patterned as fine electrodes with narrow widths because locally disconnected areas are produced by etching AgNFs. For these narrow patterns such as the bottom electrodes (width: 65 μm, space: 15 μm) of the fingerprint sensors and the source (*S*)/drain (*D*) (width: 15 μm) of pressure-sensitive FETs, random networks of AgNWs (average length of AgNWs: 30 ± 7 μm, diameter: 20 ± 5 nm) were successively electrosprayed on top of the electrospun AgNF networks using a suspension of AgNWs (B 424-1, Nanopyxis) and a nozzle (nozzle inner diameter: 0.64 mm). These sprayed AgNWs can bridge across the locally disconnected, open areas of AgNF networks to preserve the resistance of these narrow electrode patterns. After photolithographically patterning the AgNF–AgNW hybrid networks as transparent electrodes, a sputter was used to deposit a 2-μm-thick SiO_2_ layer, which was then patterned as the dielectric layer of the fingerprint sensors while uncovering the channel part of the pressure-sensitive FETs. For uniform deposition of this SiO_2_ layer on the AgNF–AgNW hybrid (r.m.s. roughness of 126 nm, Supplementary Fig. 4), the substrate was rotated at the speed of 36˚/s during SiO_2_ deposition with the deposition rate of 0.1 nm/s. As shown in Supplementary Fig. 5, this SiO_2_ layer was deposited on the AgNF–AgNW hybrid structure without any significant voids or delamination. To form a temperature sensor, a 300-nm-thick layer of poly(3,4-ethylenedioxythiophene):polystyrene sulfonate (PEDOT:PSS) was patterned on the SiO_2_ surface, instead of the AgNF–AgNW hybrid (Supplementary Fig. 6). After depositing the 2-μm-thick SiO_2_ layer again with the opening of the channel part of the pressure sensor, an amorphous layer of indium gallium zinc oxide (IGZO) (thickness: 25 nm) was sputtered as the semiconducting channel (between *S*/*D*) of the pressure-sensitive FETs. Separately, transparent electrodes of AgNF–AgNW hybrid networks were patterned as gate electrodes of these pressure-sensitive FETs on a dielectric cover layer before spinning a silicone elastomer layer (Ecoflex 0030, thickness of 30 μm). For this cover layer, thin glass layers with varied thicknesses (100–500 μm) or transparent cellulose composite films (thickness: 100 μm, with BaTiO_3_ nanoparticles) (average diameter: 50 nm) or AgNFs (average diameter: 300 nm, average length: 220 μm) were embedded as fillers to increase the dielectric constants (*k*) of these films^17–20^. After turning over this sample, the elastomer surface was exposed by an ozone-producing ultraviolet lamp and then bonded to the SiO_2_ top layer of the fingerprint sensor sample.

### Characteristics of AgNF–AgNW hybrid electrodes

**Fig. 2:**
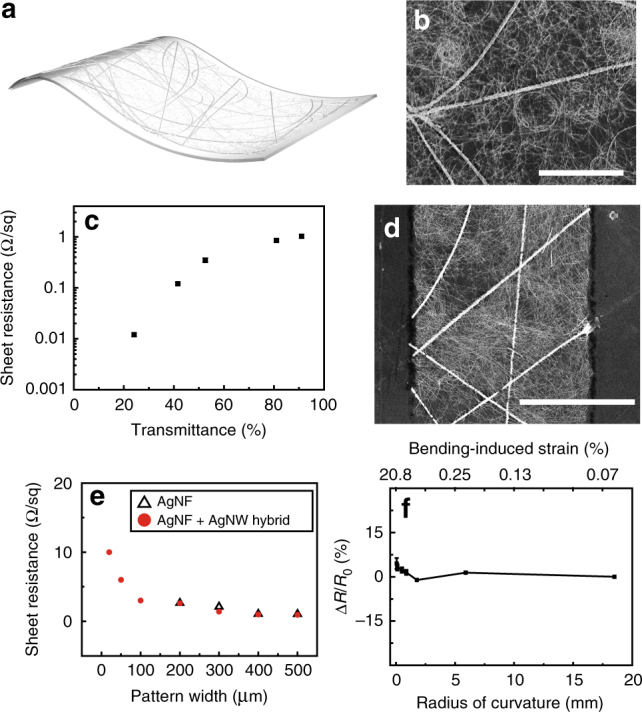
Optical, electrical, and mechanical properties of the hybrid electrode. **a** Schematic illustration of the silver nanofiber–silver nanowire (AgNF–AgNW) hybrid electrode. **b** Scanning electron microscope (SEM) of the AgNF–AgNW hybrid electrode. Scale bar, 20 μm. **c** Transmittance versus sheet resistance of the AgNF–AgNW hybrid electrode. **d** SEM image of the patterned AgNF–AgNW hybrid electrode. Scale bar is 30 μm. **e** Dependence of sheet resistance on the pattern widths of the hybrid electrode (pattern length: 500 μm). **f** Relative resistance changes as a function of the bending radius and bending-induced strain

Figure 2 presents the properties of AgNF–AgNW hybrid electrodes for the transparent fingerprint sensor array. The electrospun AgNFs have diameters of an order of magnitude larger than the diameter of AgNWs (average diameter of AgNF: 338 ± 35 nm, AgNW: 20 ± 5 nm) and are continuous to form random networks with large open spaces. Although these ultra-long AgNF networks are advantageous for obtaining low *R*_*s*_ with relatively high transparency, the large empty spaces of these networks can be disadvantageous for patterns of fine electrodes. The electrosprayed AgNWs (electrospray duration: 5 s) partially filled the vacant areas of the AgNF networks by bridging across individual AgNFs and forming conductive paths to preserve the resistance of these narrow electrode patterns (Fig. 2a, b). The area fraction of the AgNF and AgNW networks, which can be controlled by the electrospinning duration, determines *R*_*s*_ and *T* of the transparent AgNF–AgNW hybrid electrode. Both *R*_*s*_ and *T* decrease as their area fraction increases, as shown in Supplementary Fig. 7. Figure 2c presents *R*_*s*_ of the resulting AgNF–AgNW hybrid network as a function of its *T* in the visible light range (wavelength: 550 nm). As a transparent electrode, the AgNF–AgNW network formed by electrospinning for 4 s (area fraction of AgNFs: 0.034) with successive electrospraying for 5 s (area fraction of AgNWs: 0.03) exhibited a significantly low *R*_*s*_ value of 1.03 ± 0.08 Ω/sq with a transmittance of 91.04% (Supplementary Fig. 8). This optoelectronic property was superior to that of other transparent conducting materials, such as ITO (*R*_*s*_ > ~50 Ω/sq), networks of metal nanowires (*R*_*s*_ > ~20 Ω/sq), or chemical vapor deposition-synthesized graphene (*R*_*s*_ > ~100 Ω/sq for undoped cases). In addition, *R*_*s*_ of the AgNF–AgNW hybrid electrode decreased further to ~0.012 ± 0.0008 Ω/sq with *T* of 24.11% by increasing their densities. AgNF–AgNW hybrid networks can be photolithographically patterned using wet etching without any significant increase in the resistance, compared to the cases where only singular components of AgNWs or AgNFs were used with no hybrid structure (Fig. 2d, Supplementary Fig. 9, and Supplementary Table 1). Also, we performed the adhesion test of the AgNF–AgNW networks on a PI film by immersing them for 5 min each in deionized (DI) water, acetone, isopropyl alcohol, and tetramethylammonium hydroxide-based photoresist developer (AZ 300 MIF). The sheet resistance (*R*_*s*_) and area fraction of these AgNF–AgNW networks degraded negligibly, which suggested that the AgNF–AgNW networks had good adhesion and could withstand the conventional photolithography process (Supplementary Table 2). Patterning the percolated networks can significantly change their *R*_*s*_ (Fig. 2e). For example, the *R*_*s*_ of the AgNF network (without hybrid forms) showed a large variation by changing the widths of patterns because locally disconnected areas were produced by etching NFs, and became nonconductive for a width below ~200 μm, which was similar to the vacant space of its network. This *R*_*s*_ dependence on width can yield undesirable local changes in the resistance of circuits, and hence can limit the use of AgNFs in the design of compactly integrated circuits that require fine electrode geometries. On the other hand, the AgNF–AgNW hybrid structure exhibited a negligible dependence of *R*_*s*_ on the pattern widths, and it had a significantly low *R*_*s*_ even for narrow patterns with widths <100 μm. This enabled the fine patterns of transparent electrodes required in fingerprint sensors that need to detect the period between the ridges and valleys of human fingers (~100 μm)^9,21,22^. Figure 2f shows the relative change in resistance of the AgNF–AgNW hybrid electrode that was formed on a c-PI film (thickness: 25 μm) as a function of the radius of curvature and the corresponding bending-induced strain (*ε*). No significant change in resistance was observed even when the electrode was bent to a radius of curvature as small as 60 μm (*ε* < 20.8%), which indicates the superb flexibility of AgNF–AgNW networks. The stretchability of a hybrid electrode was measured by forming AgNF–AgNW networks on a polydimethylsiloxane (PDMS) sheet, as shown in Supplementary Fig. 10. Stretching this sample up to 90% (in tensile strain) uniaxially resulted in a slight increase in *R*_*s*_ from the initial *R*_*s*_ of ~1.03 Ω/sq. Also, to investigate its durability against repetitive stretching and releasing, the *R*_*s*_ of this sample was measured during repetitive deformation (15,000 cycles of 70% strain stretching). Supplementary Fig. 10 shows that *R*_*s*_ remained almost constant throughout this cyclic test, indicating its excellent reliability against such deformation.

### Characteristics of the fingerprint sensor array

**Fig. 3:**
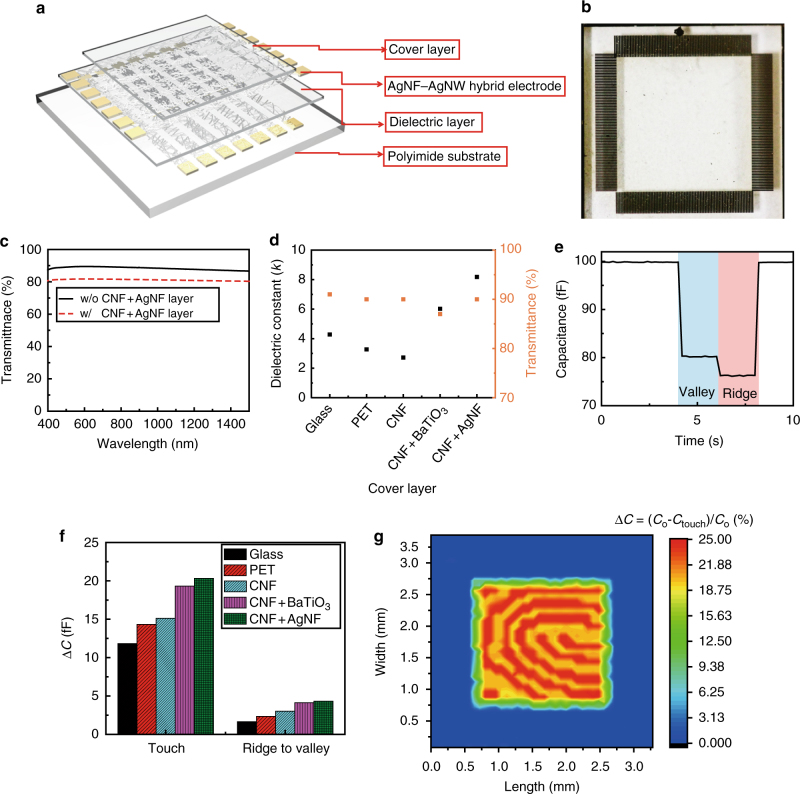
Flexible, transparent fingerprint sensor. **a** Schematic illustration of the fingerprint sensor structure. **b** Photograph of the transparent fingerprint sensor. Scale bar, 1 mm. **c** Optical transmittance spectrum of the transparent fingerprint sensor (w/ and w/o cellulose nanofiber (CNF)–AgNF layer, thickness: 100 μm). **d** Optimized transmittance at the wavelength of 550 nm and a dielectric constant at a frequency of 1 MHz with various cover layer films. **e** Real-time single-cell capacitance change according to the ridge–valley position change. **f** Capacitance change depends on the fingerprint ridge and valley with various cover layers. **g** Captured 2 mm × 2 mm fingerprint pattern

Based on the AgNF–AgNW hybrid electrode’s superb optoelectronic property and its patternability, a transparent and flexible fingerprint sensor array was demonstrated. Figure 3a shows the structure of this array, which consisted of the driving and sensing electrodes using the AgNF–AgNW networks, and a transparent dielectric layer (sandwiched between these electrodes) of 2-μm-thick SiO_2_. The top surface of this sensor array can be passivated with a transparent cover layer of glass, polyethylene terephthalate (PET), or a cellulose film with varied thicknesses. The static capacitance (*C*) between two parallel electrodes is calculated as $C = \varepsilon \frac{{wl}}{d} + \frac{{2\pi \varepsilon l}}{{\log \left( {\frac{{4d}}{h}} \right)}}$, where *ε* is the permittivity of the dielectric layer (SiO_2_) between two parallel electrodes, *w* is the width of the overlapped area, *l* is the length of the overlapped area, *d* is the thickness of the dielectric layer, and *h* is the thickness of the electrode^22,23^. Based on this equation, *C* of the designed fingerprint sensor is 100 fF (*l*: 65 μm, *w*: 65 μm, and *d*: 2 μm). Figure 3b presents a photograph of this fingerprint sensor array, and Supplementary Fig. 9 shows optical micrographs (dark field) of this sample as magnified images. This transparent sensor array has 80 × 80 electrodes or 6400 capacitor nodes in an area of 6.4 mm × 6.4 mm, which translates to 318 CPI and therefore satisfies FBI criteria. As shown in Fig. 3c, this resulting array (without the CNF + AgNF cover layer) exhibited a high transparency of 89.05% in the visible light range. Supplementary Fig. 11 presents transparency with different cover layers (PET, glass, CNF + BaTiO_3_, and CNF). This sensor array detects fingerprints by measuring the capacitance at each addressable electrode, and thus, the dielectric constant (*k*) of the protective cover layer, which is located between the active surface of the fingerprint sensor and the finger, is directly related to the fingerprint sensor sensitivity^24–26^. Glass has high optical transmittance (~90.54%), an outstanding mechanical reliability, and relatively high dielectric constants (e.g., gorilla glass, *k* = 7.2)^27^. Although glass has been used extensively as the protective cover layer for capacitive-type fingerprint sensors, its fragility limits its use for flexible devices. Also, conventional, transparent plastic films, such as PET (*k* = 3.1), polyethylene (PE) (*k* = 2.2), PI (*k* = 3.4), and polycarbonate (PC) (*k* = 2.9), exhibit a relatively low *k* and modest mechanical properties for withstanding wear and scratch damage. As such, they are also not suitable for the cover layer of fingerprint sensors, which requires a high *k* and outstanding mechanical durability as well as a high *T*.

In this study, we tested five different transparent films: glass slide, PET, cellulose nanofibers (CNFs) film, the CNF film embedded with BaTiO_3_ nanoparticles (CNF + BaTiO_3_, the content of the TiO_2_ nanoparticle: 1 wt%, and average size: 25 nm), and the CNF film embedded with AgNFs (CNF + AgNF, the content of AgNFs: 1.2 wt%, average length of AgNFs: 200 ± 20 μm, and average diameter of AgNFs: 380 ± 35 nm)^17,20^, as the cover layer (thickness of these layers: 100 μm) of the fingerprint sensor array. Although CNF films are advantageous due to their high transparency and good mechanical flexibility and durability, the low dielectric constants of pristine CNF films (*k* = 1.4–3.0) still limit their use as the cover layer of this fingerprint sensor array^19,20,28–30^. Embedding nanofillers of metals (AgNFs) or ceramics (BaTiO_3_ nanoparticles) into the CNF film can significantly increase *k*^17,31–33^. Figure 3d and Supplementary Fig. 12 compare the *T* (at 550 nm) and *k* values of these cover layers. Here, *k* values were measured at 1 MHz, which is the operating frequency of the transparent fingerprint sensor array. Among these five different cover layers, the CNF film with AgNFs (CNF + AgNF) presented the highest *k* value of 9.2 with good transmittance of ≈90%. Figure 3e shows the change in the capacitance (Δ*C*) of a single cell from the sensor array with this CNF + AgNF layer at an operating frequency of 1 MHz (untouched static capacitance, 100 fF) by touching a finger on the top surface of this cover layer and then slipping sideways as shown in Supplementary Fig. 13. When the valley of the fingerprint was touched onto the fingerprint sensor, the capacitance was reduced from 100 ± 0.08 to 80 ± 0.12 fF. Consecutively, the fingerprint was slid, and the ridge of the fingerprint was located with reducing the capacitance from 80 ± 0.12 to 76 ± 0.09 fF. In addition, we measured Δ*C* using these five different films covering the fingerprint sensor array. Among these samples, the highest *k* case (the fingerprint sensor using a CNF + AgNF cover layer) presented the largest capacitance change (Fig. 3f). Figure 3g shows the two-dimensional fingerprint mapping results using a fake fingerprint (size: 2 mm × 2 mm). This sensor array recognized a Δ*C* (from the ridge to the valley of the fingerprint) of 4.03%, and the difference between the fake fingerprint pattern and the mapping image from the sensor array was negligible.

### Entire circuit system for the fingerprint sensor array

**Fig. 4:**
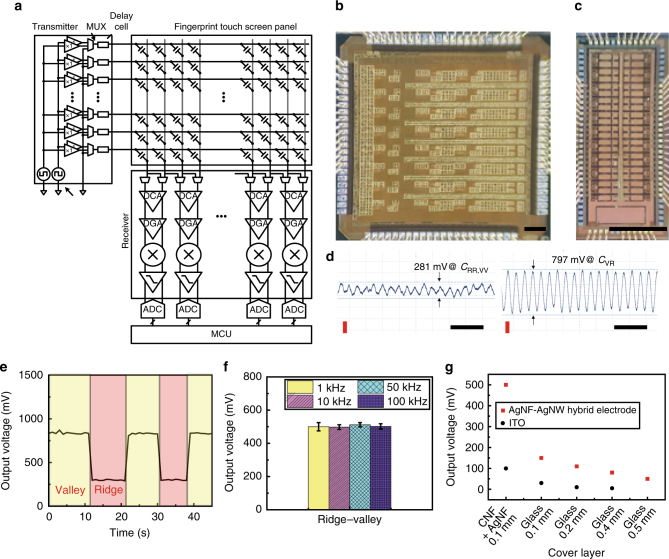
Custom-designed fingerprint sensor readout circuit. **a** Block diagram of the fingerprint sensor readout circuit. **b** Fully differential receiver for fingerprint recognition. Scale bar is 250 μm. **c** High-voltage transmitter. Scale bar is 1 mm. **d** Measurement result of the sensing ridge to the valley indicated the analog voltage outputs of the proposed IC and the fingerprint TSP (1 MHz, 1 V). Output voltages are 281 and 797 mV under ridges and valleys. *Y*-axis scale bar (red) is 200 mV and the *x*-axis scale bar (black) is 2 μs. **e** Waveform output voltage change during the fingerprint sweep. **f** Output voltages of the fingerprint sensor depend on fingerprint ridges and valleys with different noise frequencies. **g** Comparison of output voltages of the fingerprint sensor using a silver nanofiber (AgNF)–silver nanowire (AgNW) hybrid versus indium thin oxide (ITO) electrodes with different thickness of glass cover layers and a cellulose nanofiber (CNF) + AgNF cover layer

Figure 4a illustrates the entire circuit system composed of this fingerprint sensor array as a touch screen panel (TSP), a driving unit, and a receiving unit. The TSP consists of the driving electrode and the sensing electrode arranged in parallel, and mutual capacitors are placed between these electrodes. When a finger touches the fingerprint TSP, the difference in the mutual capacitance occurs dependent on the difference in the depths of the ridges and valleys. This circuit system obtains the fingerprint image by sensing the mutual capacitance. The transmitters send driving signals to the driving electrodes in a time-division manner. The transmitter is composed of a reference generator, a selection block, a buffer, and a multiplexer (MUX). A receiver is designed based on a fully differential circuit, which has the advantage of detecting low differences in capacitance. A fully differential receiver is composed of an MUX, a differential charge amplifier (DCA), a differential gain amplifier (DGA), a multiplier, a low-pass filter (LPF), an analog-to-digital converter (ADC), and a microcontroller unit (MCU). The output of DCA is proportional to the difference in the adjacent capacitors, and a DGA amplifies the output of a DCA. A multiplier and a LPF block any noise signals, and the ADC converts the analog signals to digital signals. In this way, a fingerprint image can be obtained by processing the digital signal.

Figure 4b, c presents optical micrographs of a fully differential receiver for fingerprint recognition and a high-voltage transmitter. Figure 4d shows the measurement results using the fingerprint system. These results indicate the analog outputs of the proposed IC and the fingerprint TSP. When a differential receiver senses a mutual capacitance under the ridges or the valleys (*C*_RR_, *C*_VV_), the output of the receiver is 281 mV as shown in Fig. 4d. When a differential receiver senses a mutual capacitance under the ridges and the valleys (*C*_VR_), the output of the receiver is 797 mV as shown in Fig. 4d. Comparing the adjacent capacitance of the whole fingerprint TSP, it is possible to make a capacitive contour map. Figure 4e shows the output waveform of the fingerprint sensor. The output voltage of this sensor is ~0.30–0.83 V with a frequency of 1 MHz. A fingerprint was slipped on the fingerprint sensor with 10 μm/s and the output voltage was measured. A driving circuit applied the signal with 1 V at 1 MHz to the electrode, and then 500 ± 4 mV of the output voltage was obtained according to the positions of the ridges and valleys of the fingerprint. In order to integrate this transparent fingerprint TSP on a display, it is necessary to have reliable operations of this TSP in high-frequency ranges to avoid noise from the display (<200 kHz) in mobile applications. Figure 4f presents the difference in the output voltages under the ridges and the valleys against various noise signals. This transparent fingerprint TSP operated reliably at a high frequency of 1 MHz with negligible degradation in its performance against typical noise signals (1, 10, 50, and 100 kHz) from mobile devices, due to the significantly low *R*_*s*_ of AgNF–AgNW hybrid electrodes. In comparison, another fingerprint TSP was fabricated with identical structures where transparent ITO (thickness: 100 nm, *R*_*s*_: 30 Ω/sq) and AgNWs (*R*_*s*_: 11.3 Ω/sq) were used as the driving electrode and the sensing electrode, instead of the AgNF–AgNW hybrid networks. As shown in Fig. 4g and Supplementary Fig. 14, the fingerprint TSP using the AgNF–AgNW hybrid had the largest difference in output voltages under the ridges and the valleys, while the fingerprint TSP (covered by a 500-μm-thick glass layer) using AgNWs or ITO could not distinguish the ridges and valleys.

### Multiplexed fingerprint sensor array

**Fig. 5:**
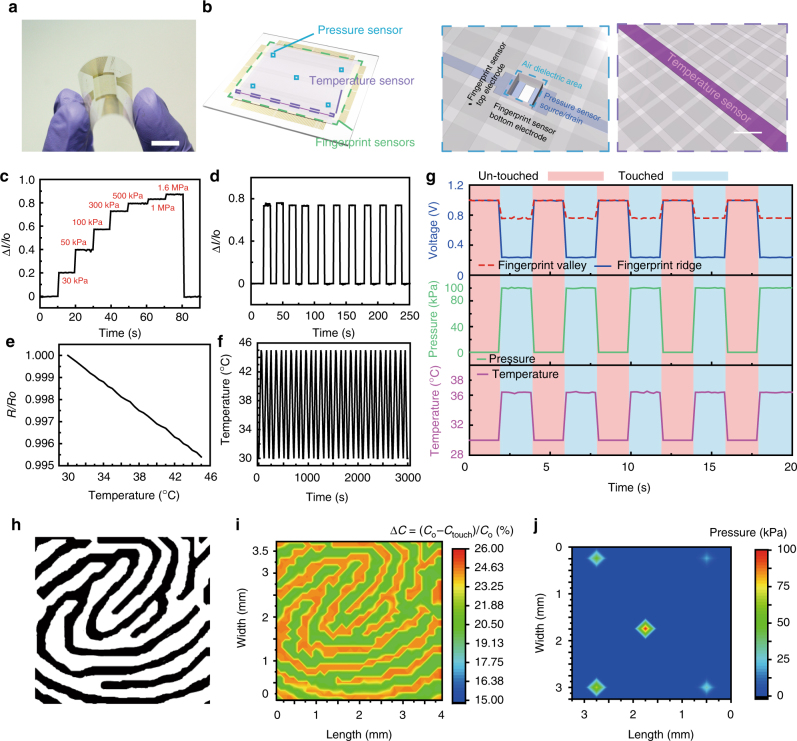
Fully integrated multiplexed fingerprint sensor. **a** Optical micrograph multiplexed fingerprint sensor. Scale bar, 1 cm. **b** Schematic illustration of a multiplexed fingerprint sensor. **c**, **d** Real-time measurements of normalized drain current changes for applied pressure at *V*_*D*_ = 10 V and *V*_*G*_ = 30 V. Different amounts of pressure are applied one by one, sequentially representing step-like features (**c**). Pressure (100 kPa) is loaded and unloaded repeatedly to evaluate stable and reliable operation (**d**). **e** Temperature versus normalized resistance. **f** Temperatures (30–45 ℃) are loaded and unloaded 30 times to evaluate stable and reliable operation. **g** Real-time sensing graph of the multifunctional fingerprint sensor array. **h** Scan image of the real fingerprint. **i** Two-dimenstional (2D) mapping image of the relative changes in the capacitance of the fingerprint sensor upon the touch of human finger. **j** Color gradation contour plot of the resultant signals (Δ*I*_*D*_/*I*_*o*_) detected from five different pressure sensors by the single touch of a finger

As the level of device integration continually increases, the multifunctionality of sensors becomes increasingly important for mobile smart devices. Figure 5 describes the integration of this fingerprint sensor array with tactile pressure sensors and skin temperature sensors, all of which have transparent and flexible forms, to enable the detection of a finger pressing on the display. This facilitates the removal of activation buttons on smart devices. Additionally, the ability to recognize artificial fingerprints improves security. Figure 5a presents a photograph of this integrated, multifunctional sensor array. All transparent sensors for the fingerprint, pressure, and temperature are located in the central transparent region inside the outer bezel areas to interconnect these sensors to the readout circuit using Cr/Au electrodes. Figure 5b shows schematic layouts of this array. In the case of pressure sensors, we fabricated pressure-sensitive FETs with local air gaps as a dielectric layer between the channel (IGZO) and the top-gate electrode, for good electrical properties and high reliability under ambient conditions, due to the clean interface between IGZO and air (supplementary information)^34^. Here, an elastomeric dielectric layer (Ecoflex 0030, thickness: 20 μm) was located on the 4-μm-thick air gap (below a 100-μm-thick, transparent cover layer of the CNF + AgNF hybrid), and its thickness can decrease by applying pressure with increasing the capacitance of IGZO FET. Five pressure sensors were located on the four corners and at the center of the fingerprint sensor array. The thickness of this air gap on the channel part reduces with an increase in the capacitance of the gate–air dielectric–IGZO structure when this pressure-sensitive FET is pressed by a normal mechanical force, which increases the *S*/*D* current (*I*_*D*_) of this FET (Supplementary Fig. 16) Supplementary Fig. 17 shows a SEM image of a pressure sensor that was located between neighboring electrodes of fingerprint sensors. *I*_*D*_ versus top-gate bias (*V*_*G*_) characterization of this FET was measured at an ambient condition, and its representative transfer and output curves are presented in Supplementary Fig. 18a, b. This air-dielectric FET shows the n-channel behavior, with the mobility, on/off ratio, and threshold voltage of 69.7 cm^2^/V/s, 1.11 × 10^6^, and 10 V, respectively, in a linear regime. The air-dielectric layer becomes thinner, thus resulting in a higher *I*_*D*_ with increasing pressure. Supplementary Fig. 19 presents the plot of the normalized change in drain current (Δ*I*_*D*_*/I*_*o*_) versus applied pressure, extracted at *V*_*D*_ = 10 V and *V*_*G*_ = 30 V. The detectable maximum pressure value is ~1.6 MPa and Δ*I*_*D*_ saturates beyond this pressure range, in which the sensitivity is calculated as ~1.78 × 10^−3^ kPa^−1^ at a lower pressure regime (below 350 kPa) and ~9.65 × 10^−5^ kPa^−1^ at a higher pressure regime (above 350 kPa). This pressure sensor is capable of detecting a wide range of pressure, which is of significant importance, representing a potential beyond the range of the gentle touch of human fingers to object manipulations (from 10 to 100 kPa)^35,36^. When pressure was applied on this device, the real-time detection curve of Δ*I*_*D*_/*I*_*o*_, as shown in Fig. 5c presents distinctive step-like features. Figure 5d shows the recovery behavior in pressure sensing with negligible hysteresis during repeated loading–unloading tests with a pressure of 300 kPa. This pressure sensor operates with a response time of 32 ms and a recovery time of 56 ms.

PEDOT:PSS was used as a transparent, temperature-sensitive material, and Supplementary Fig. 20 presents a SEM image of this PEDOT:PSS pattern (as a temperature-sensitive resistor) integrated with the fingerprint sensor array. The initial resistance value (*R*_*o*_) of this temperature sensor was 8.5 kΩ, and temperature modulated its normalized resistance (*R/R*_*o*_) linearly in the range between 30 ℃ and 45 ℃ (Fig. 5e). PEDOT:PSS typically exhibits a negative temperature coefficient (NTC)^37–39^, and the average temperature coefficient of resistance (TCR) of this sensor was 0.03% per ℃. Furthermore, to observe the reliability of temperature sensing, the resistance change of the temperature sensor was measured and converted to temperature. Temperature was controlled by a hot plate (30–45 ℃), and the temperature sensor exhibited linear NTC behavior in this temperature range. The hysteresis level was negligible. As shown in Fig. 5f, the temperature can be measured reliably and repeatably for 30 times in a cyclic test.

Figure 5g presents representative graphs for the simultaneous detection of a fingerprint, tactile pressure, and skin temperature using this transparent and flexible device for a series of finger touches with a 100-μm-thick, transparent cover layer of the CNF + AgNF hybrid. For simultaneous sensing, fingerprint sensors were connected to the circuit system using a peripheral connecting device. At the same time, the same peripheral connecting device and the pressure and temperature sensors were connected to two sourcemeters, the system switch, and the relay card, as shown in Supplementary Fig. 21. Here, the blue and red lines show the change in the output voltage of the fingerprint sensor (including the elastomeric layer) at ridge and valley positions, respectively. When the finger touched this device, an additional voltage drop of about 500 mV was generated in the ridge area, compared to the valley area. Also, the pressure-sensitive FETs monitored the tactile pressure from touching a finger repeatedly for five times (~100 kPa on the green line of Fig. 5g), and the temperature sensor detected the temperature of the finger skin each time the finger made contact with it (the purple line). Figure 5h, i shows an original image of a human fingerprint and the image scanned from this fingerprint sensor array, respectively. The differences in the capacitance between the ridges and valleys were about 4.03%, and the pattern of the original fingerprint and its scanned result matched with negligible deviations. Furthermore, the multiple array of pressure-sensitive FETs was located with a spacing of 1.3 mm inside this fingerprint sensor array, and hence the singular touch of a finger pressed multiple pressure sensors simultaneously. For example, Fig. 5j shows a color gradation contour plot of the resultant signals (Δ*I*_*D*_/*I*_*o*_) detected from five different pressure sensors by the single touch of a finger. When these five different FETs were pressed selectively, the signals (Δ*I*_*D*_/*I*_*o*_) only changed according to the corresponding sensor position (Supplementary Fig. 22). In order to measure the flexibility of this transparent sample with the multifunctional sensor array, it was wrapped on various cylindrical supports with different curvatures. Supplementary Fig. 23 shows the relative difference in the capacitance change between the ridges and valleys measured from a fingerprint sensor as a function of bending-induced strain. There was no significant change during bending to a radii of curvature as small as 3.1 μm.

## Discussion

In this paper, we described the fabrication of a transparent and flexible fingerprint sensor array with multifunctional detection of finger pressure and skin temperature using AgNF–AgNW hybrid networks as high-performance transparent electrodes. The high resolution of this fingerprint sensor array (318 CPI) sufficiently satisfies the criteria set by the FBI for extracting fingerprint patterns, and its good transparency (89% in the visible light regime) enables its integration into a display. The sensing capability, in terms of capacitance variation (between a ridge and a valley) is up to 17 times better than that of an identical sensor structure using conventional ITO electrodes. Furthermore, the low *R*_*s*_ of the AgNF–AgNW hybrid electrodes can drive this sensor array at 1 MHz reliably to handle typical noise from mobile devices or displays. The demonstration of its integration with pressure and temperature sensors, all of which had transparent and flexible forms, indicates the potential for replacement of the activation button on smartphones. Additionally, the ability to recognize artificial fingerprints further improves security.

## Methods

### Formation of AgNF–AgNW hybrid electrodes

We used an electrospinning process to fabricate a continuous network of Ag nanofibers (AgNFs) with an average diameter of 338 ± 35 nm using a suspension of Ag nanoparticles (NPK, Korea; average diameter: 40 ± 5 nm; solvent: ethylene glycol; concentration = 50 wt%) as an ink. The electrospinning height was 15 cm, the applied voltage between the nozzle tip and the ground was 11.5 kV, and the inner and outer diameters of the nozzle were 0.33 and 0.64 mm, respectively. The environmental temperature and relative humidity were 17 °C and 4%, respectively. The electrospun fibers were annealed at 150 °C for 30 min in air (relative humidity: ~25%). AgNWs (Nanopyxis Co. Ltd.) with an average diameter of 30 (±5) nm and length of 25 (±5) mm which were dispersed in DI water (3 mg/ml) were electrosprayed on top of the AgNF random network. The electrospraying height was 15 cm, the applied voltage between the nozzle tip and the ground was 9.5 kV, and the diameters of the nozzle were 0.33 mm.

### Formation of high-k CNF films

A total of 2,2,6,6-tetramethyl-1-piperidine-1-oxyl (TEMPO)-oxidized CNFs (0.3 wt%) about 20 nm in diameter and 1-micron long (University of Maine, Orono, ME, USA) were used to prepare a high-k CNF film. To fabricate a high-k and transparent CNF film, BaTiO_3_ nanoparticles (Sigma Aldrich) and AgNFs were mixed in an aqueous suspension of CNFs (0.3 wt%) with various concentrations, followed by vacuum filtration. The obtained CNF film was thoroughly dried by hot pressing at 60 °C for 10 h, under the pressure of 10 MPa, and was then peeled off from the filter. Next, an epoxy-based hard polymer (SU-8, Microchem) was coated by the dip-coating method and the CNF film was obtained.

### Characterization of fingerprint sensors

The capacitance changes of the fingerprint sensor were measured by a probe station (Keithley 4200-SCS and Agilent E4980A). Capacitance measurements were conducted at 1-MHz frequency with a 1-V AC signal using an Agilent E4980A Precision LCR Meter. When using a fingerprint recognition IC for fingerprint detection, a transmitter IC sends 1-MHz and 1-V AC signals to the driving electrodes of the fingerprint TSP. A receiver IC receives current from the sensing electrodes which is proportional to the mutual capacitor of the fingerprint TSP and converts these current signals to the voltage signals. By comparing these voltage signals, it is possible to make a fingerprint image. For artificial noise input test, typical noise signals (1, 10, 50, and 100 kHz, 1 V) were applied to the driving electrodes with fingerprint operation AC signal (1 MHz, 1 V) using an independent signal generator (Keysight 33520B). Output voltages with different noise signals were measured at the sensing electrodes which were connected to the receiver IC circuit.

### Characterization of pressure and temperature sensors

The electrical performances such as transfer and output characteristics of the pressure sensor and resistance of the temperature sensor were characterized by a probe station (Keithley 4200-SCS). Pressure was applied and measured by a motorized vertical test stand (Mark-10 ESM301) in combination with a force gauge (Mark-10 M5-2). Heat was applied by a hot plate. To test the pressure- and temperature-sensing performances, a homemade measuring system was built to collect electric signals when the device was under applied force and heat. For the measurements of the pressure distribution on five pressure sensors and temperature, two sourcemeters (Keithley 2400), a system switch (Keithley 3706), a relay card (Keithley 3723), and peripheral devices were used. The output signals were exhibited using the Labview-based programmed software.

### Data availability

Data supporting the findings of this study are available within the article and its supplementary information files and from the corresponding author upon reasonable request.

## Electronic supplementary material

Supplementary Information
