# Supplementary material for: Transparent and flexible fingerprint sensor array with multiplexed detection of tactile pressure and skin temperature
Source: Nat Commun. 2018 Jul 3;9:2458. doi: 10.1038/s41467-018-04906-1 (PMC6030134; doi:10.1038/s41467-018-04906-1)
Supplement: Supplementary file 1 — Supplementary Information [file 41467_2018_4906_MOESM1_ESM.docx]

**Supplementary Information**

**Transparent and Flexible Fingerprint Sensor Array with Multiplexed Detection of Tactile Pressure and Skin Temperature**

Byeong Wan An^1^, Sanghyun Heo^2^, Sangyoon Ji^1^, Franklin Bien^2★^, Jang-Ung Park^1★^

*^1^ School of Materials Science and Engineering, Samsung Display-UNIST center, Wearable Electronics Research Group, Ulsan National Institute of Science and Technology (UNIST), Ulsan Metropolitan City, 689-798, Republic of Korea*

*^2^ School of Electrical Engineering, Samsung Display-UNIST center, Ulsan National Institute of Science and Technology (UNIST), Ulsan Metropolitan City, 689-798, Republic of Korea*

^★^*e-mail:* jangung@unist.ac.kr *or* bien@unist.ac.kr

This PDF file includes:

Methods

Supporting Figures 1-23

Supporting Table 1 and 2

**Supplementary Methods**

**Fabrication of AgNF-AgNW random network electrodes**

We used an electrospinning process to fabricate a continuous network of Ag nanofibers (AgNFs) with an average diameter of 338±35 nm using a suspension of Ag nanoparticles (NPK, Korea; average diameter: 40±5 nm; solvent: ethylene glycol; concentration=50 wt.%) as an ink. The electrospinning height was 15 cm, the applied voltage between the nozzle tip and the ground was 11.5 kV, and the inner and outer diameters of the nozzle were 0.33 and 0.64 mm, respectively. The environmental temperature and relative humidity were 17 °C and 4%, respectively. The electrospun fibers were annealed at 150 °C for 30 min in air (relative humidity: ~25%). AgNWs (Nanopyxis Co. Ltd,) with average diameter of 30 (±5) nm and length of 25 (±5) mm which were dispersed in deionized water (3 mg ml^-1^) was electrosprayed on top of the AgNF random network. The electrospraying height was 15 cm, the applied voltage between the nozzle tip and the ground was 9.5 kV, and the diameters of the nozzle were 0.33 mm.

**Fabrication of high-*k* CNF films**

2,2,6,6-tetramethyl-1-piperidine-1-oxyl (TEMPO)-oxidized CNFs (0.3 wt%) about 20 nm in diameter and 1 micron long (University of Maine, Orono, ME, USA) were used to prepare high-k CNF film. To fabricate high-k and transparent CNF film, TiO_2_ nanoparticles (Sigma Aldrich) and AgNFs were mixed in an aqueous suspension of CNFs (0.3 wt%) with various concentrations, followed by vacuum filtration. The obtained CNF film was thoroughly dried by hot pressing at 60 °C for 10 hr, under the pressure of 10 MPa, and was then peeled off from the filter. Next, an epoxy-based hard polymer (SU-8, Microchem) was coated by dip-coating method and CNF film was obtained.

**Fingerprint sensor characterization**

The capacitance changes of the fingerprint sensor were measured by a probe station (Keithley 4200-SCS and Agilent E4980A). Capacitance measurements were conducted at the frequency of 1 MHz with 1 V AC signals using an Agilent E4980A, Precision LCR Meter. When using a fingerprint recognition IC for fingerprint detection, the transmitters IC sends 1 MHz and 1V AC signals to the driving electrodes of the fingerprint TSP. A receiver IC receives current from the sensing electrodes that is proportional to the mutual capacitor of the fingerprint TSP and converts these current signals to the corresponding voltage signals. It is possible to make a fingerprint image by comparing these voltage signals.

**Pressure and temperature sensor characterization**

The electrical performances, such as transfer and output characteristics of the pressure sensor and resistance of the temperature sensor, were characterized by a probe station (Keithley 4200-SCS). Pressure was applied and measured by a motorized vertical test stand (Mark-10 ESM301) in combination with a force gauge (Mark-10 M5-2). Heat was applied by a hot plate. To test the pressure and temperature sensing performances, a custom-designed measuring system was used to collect electric signals when the device was under applied force and heat. For the measurements of the pressure distribution on five pressure sensors and measurements of temperature, we used two source meters (Keithley 2400), a system switch (Keithley 3706), a relay card (Keithley 3723), and peripheral devices. The output signals were exhibited using the LabVIEW-based programmed software.

**Simultaneous sensing of fingerprint, pressure, and temperature**

Using a peripheral connecting device, fingerprint sensors were connected to the circuit system, as shown in Supplementary Figure S21. At the same time, using the same peripheral connecting device, pressure and temperature sensors were connect to the two source meters (Keithley 2400), the system switch (Keithley 3706), and the relay card (Keithley 3723). When the fingerprint was applied to the fingerprint sensors, simultaneous measurements were made of the capacitance change, pressure, and temperature.

**Electrical characterization**

Electrical characterizations were conducted with the transfer and output characteristic measurement using a Keithley 4200-SCS semiconductor parametric analyzer. In figure 3b shows different sheet resistances were calculated from measured resistances by aspect ratio of patterns (*R_s_*$=R\times\frac{film width}{film length}$).

**Error bar calculation**

Error bars of relative resistance change were calculated using standard deviation equation,

*Error bar* $= \frac{\sqrt{\frac{\sum_{k=1}^{n} {(R_{k}-R_{avg})}^{2}}{n}}}{R_{o}}\times100 \left( \% \right),$

where *R_k_* is the resistance of each point, *R_avg_* is the average resistance of all analyzed points under tensile strain, n is the number of analyzed points, and *R_o_* is the initial average resistance.

**Mechanical characterization**

Electrical characterization was conducted under mechanical strain (bending and stretching) using a Keithley 4200-SCS semiconductor parametric analyzer. Induced strain was calculated using following equations.

Bending-induced strain = $\frac{F\left( Ts+Tf \right)}{2Rc}$ (*T_s_* : thickness of substrate, *T_f_* : thickiness of film, *R_c_* : bending radius)

Stretching-induced strain = $\frac{\mathrm{Ls}}{\mathrm{Lo}}\times100$ (*L_s_* : length after stretching, *L_o_* : length before stretching).

**Optical characterization**

The optical transmittance was measured using a UV-Vis-NIR spectroscopy (Cary 5000 UV-Vis-NIR, Agilent, Santa Clara, CA, USA) with a diffuse reflectance accessory, and the transmittance of the substrate was used as a baseline. The transmittance of the substrate was excluded. The transmittance at 550 nm was plotted in the manuscript.

**Adhesion test**

We performed the adhesion test to check the adhesion of the AgNF-AgNW random networks to the PI film. We immersed the AgNF-AgNW random networks on the PI film in deionized (DI) water, acetone, isopropyl alcohol (IPA), and tetramethylammonium hydroxide (TMAH)-based photoresist developer (AZ 300 MIF) for five minutes. Sheet resistance (Rs) and area fraction of the AgNF-AgNW random networks changed negligible, which suggests that the AgNF-AgNW random networks show good adhesion to the PI film. The adhesion of the AgNF-AgNW random networks on the PI film against organic solvents showed that our hybrid electrode is suitable for conventional photolithography.

Supplementray Figures


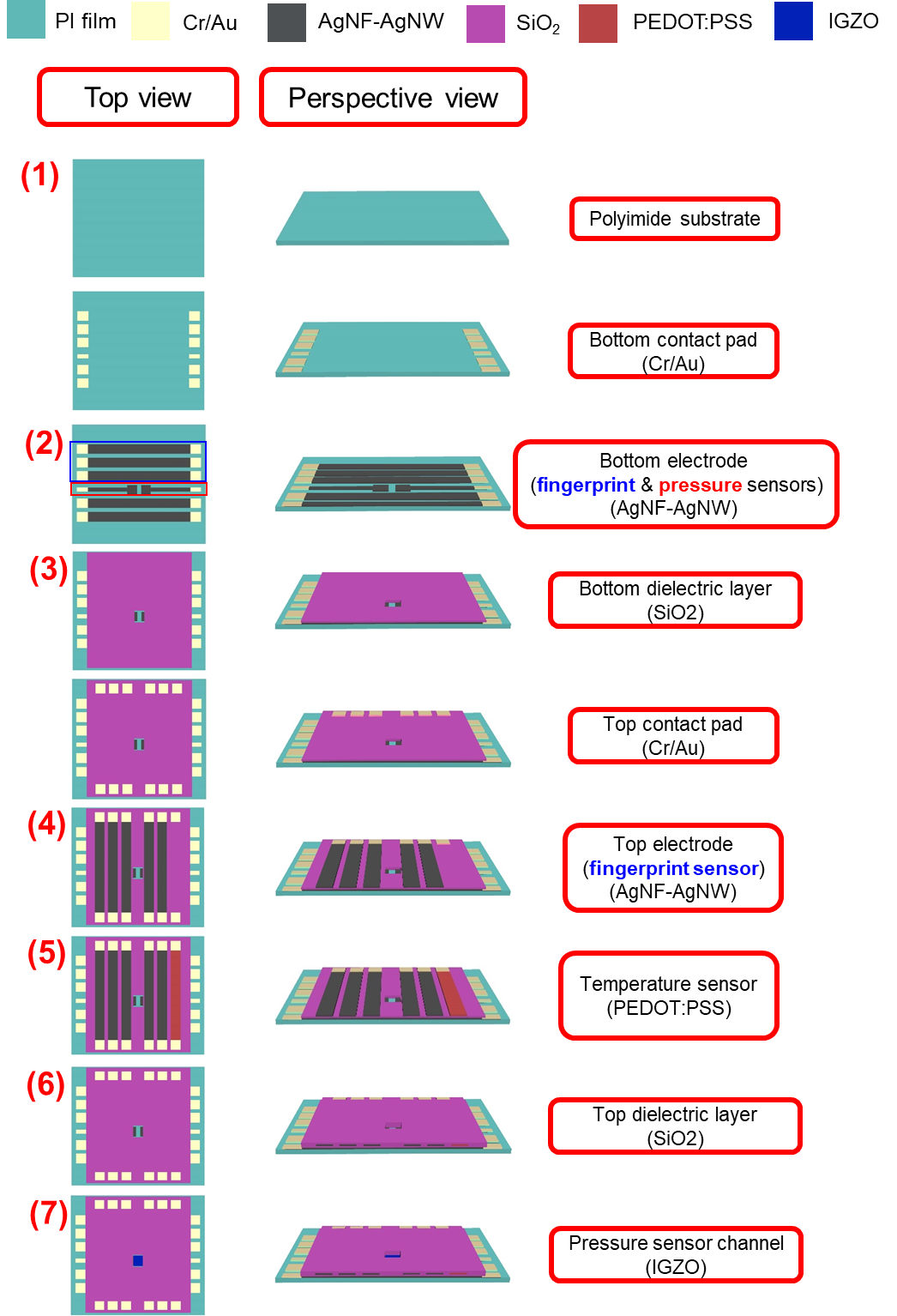


Supplementary Figure 1 │ Fabrication steps of sensors for detecting fingerprint, pressure, temperature.


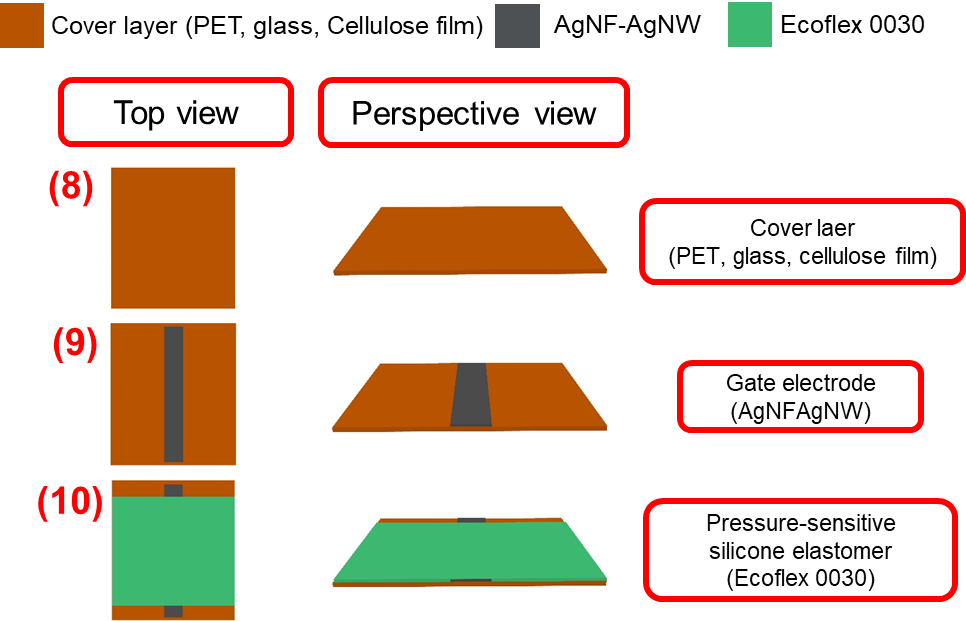


Supplementary Figure 2 │ Fabrication steps of the pressure-sensitive cover layer.


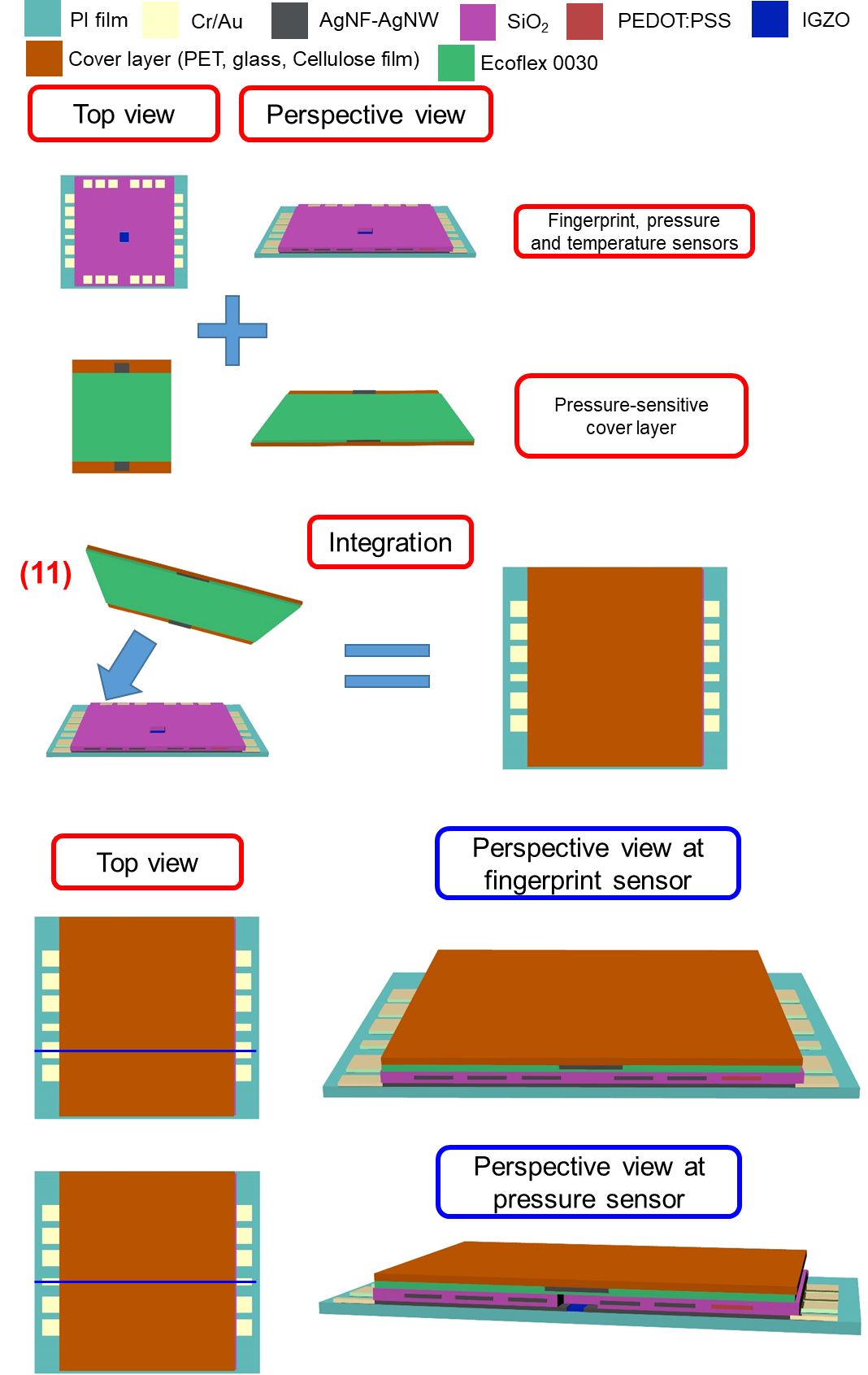


Supplementary Figure 3 │ Fabrication steps for device integration.


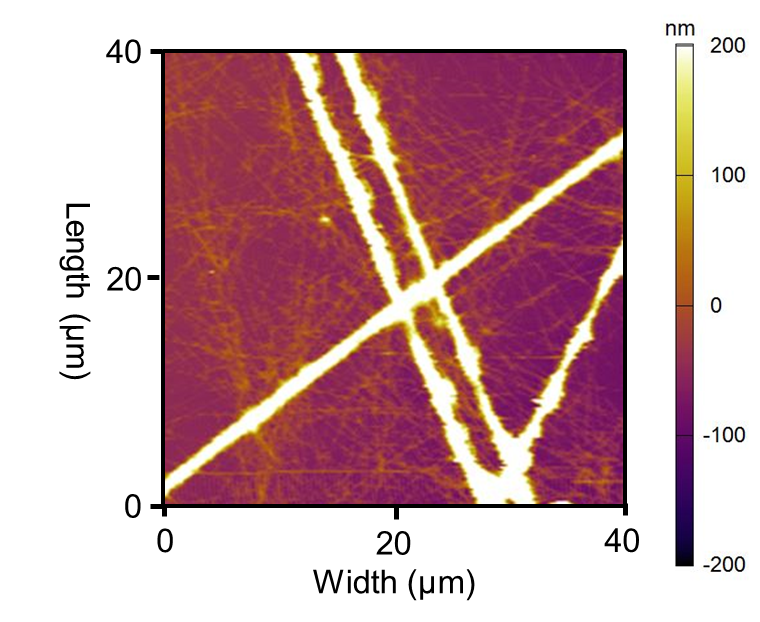


Supplementary Figure 4 │ Surface of the AgNF-AgNW hybrid electrode. AFM image of the AgNF-AgNW hybrid networks.


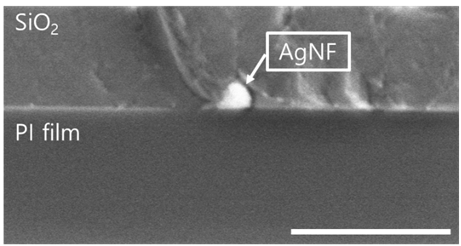


**Supplementary Figure 5** │ **Cross-section of the AgNF-AgNW.SiO_2_ layer.** Cross-section SEM image to show the interfaces of PI film / AgNF-AgNW hybrid / SiO_2_ layer. Scale bar is 2 μm.


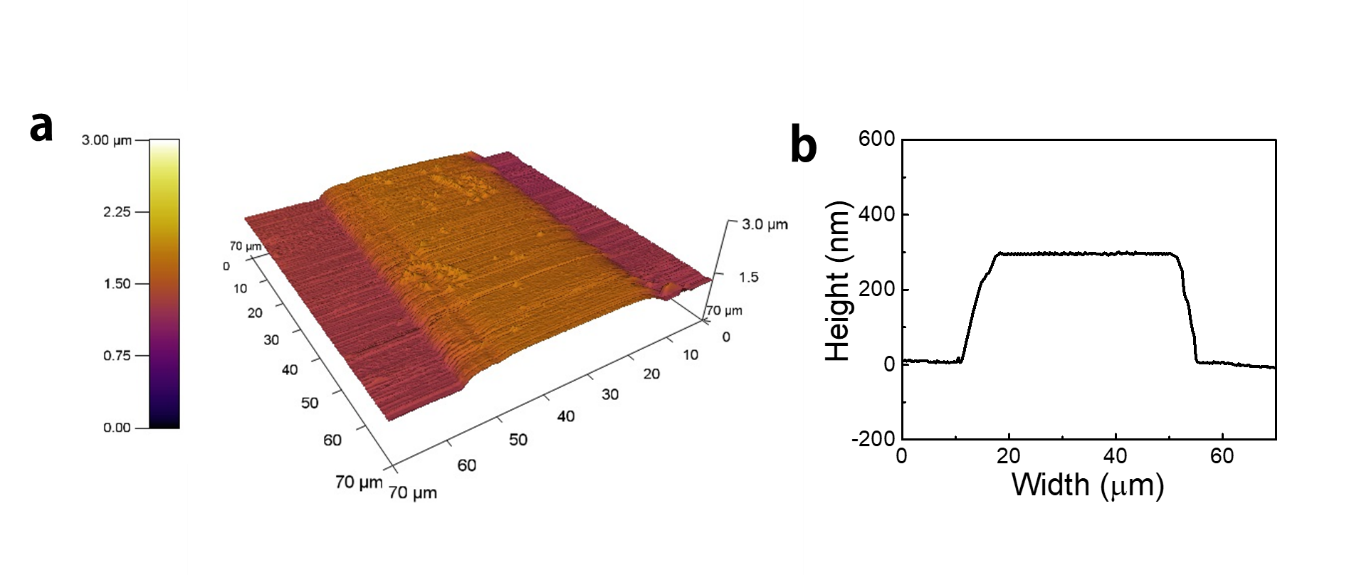
 Supplementary Figure 6 │ Thickness of PEDOT:PSS line. a, AFM image of PEDOT:PSS line. b, Cross-section profile of PEDOT:PSS line.


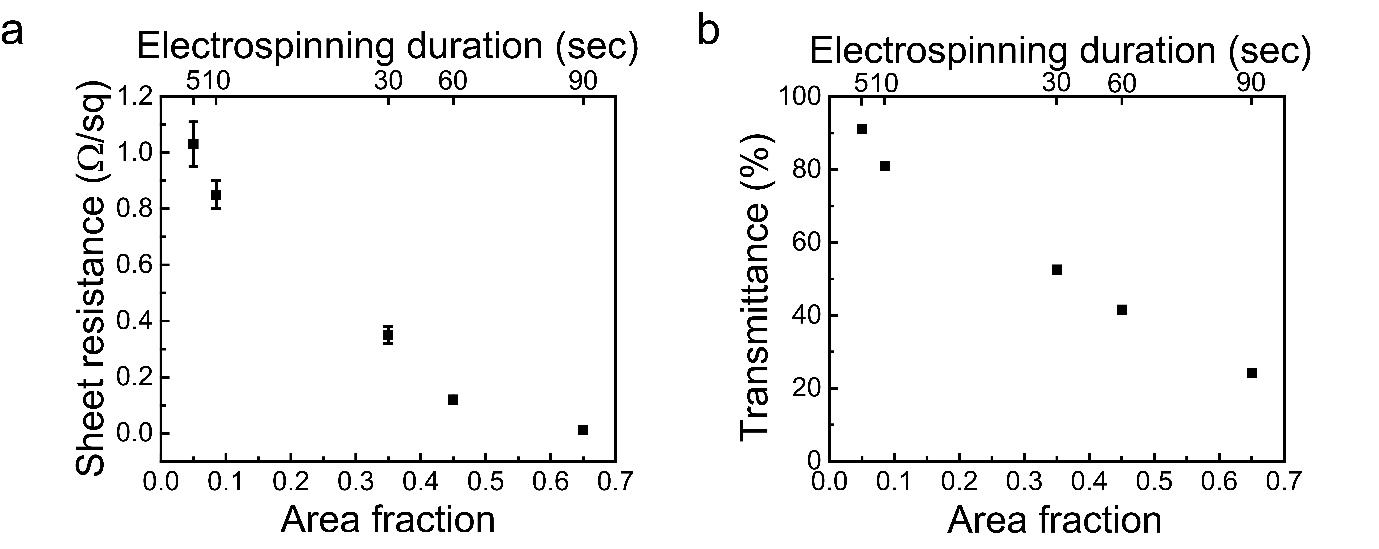


Supplementary Figure 7 │ Optical and electrical properties of the AgNF-AgNW hybrid electrode. (a) Sheet resistance and (b) transmittance as the function of area fraction and electrospinning duration. The values of error bar of the sheet resistance are standard deviation. Calculation was followed “error bar calculation” method in supplementary method.

Supplementary Figure 8 │ Transmittance as a function of wavelength for different sheet resistance of the AgNF-AgNW hybrid electrode.


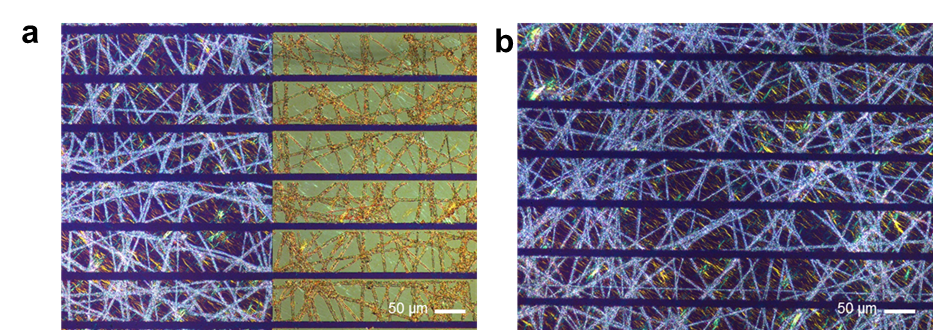


Supplementary Figure 9 │ Optical micrographs of fingerprint sensor elecrtrodes. Dark field optical micrograph of fingerprint sensor electrode with (a) contact pad and (b) without contact pad.


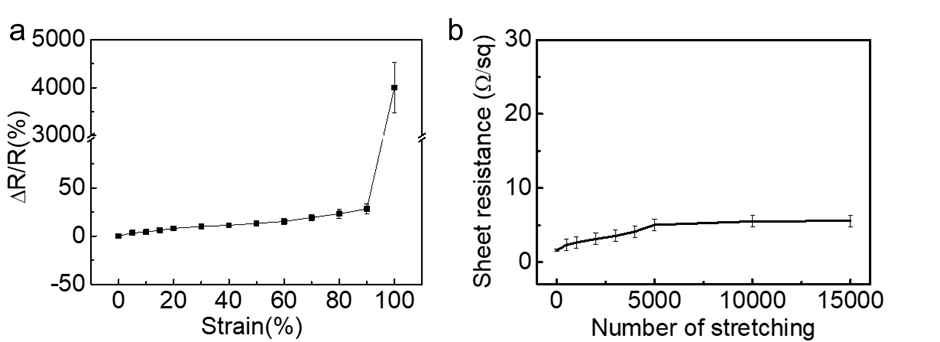


Supplementary Figure 10 │ Stretchability of the AgNF-AgNW hybrid electrode. a. Sheet resistance of the AgNF-AgNW hybrid electrode as a function of tensile strain. b. Change of sheet resistance of the AgNF-AgNW hybrid electrode for 15,000 cycles of 70%-strain stretching. The values of error bar of the resistance are standard deviation. Calculation was followed “error bar calculation” method in supplementary method.

Supplementary Figure 11 │ Transmittance of fingerprint sensor with various cover layer film (Thickness: 100 μm)


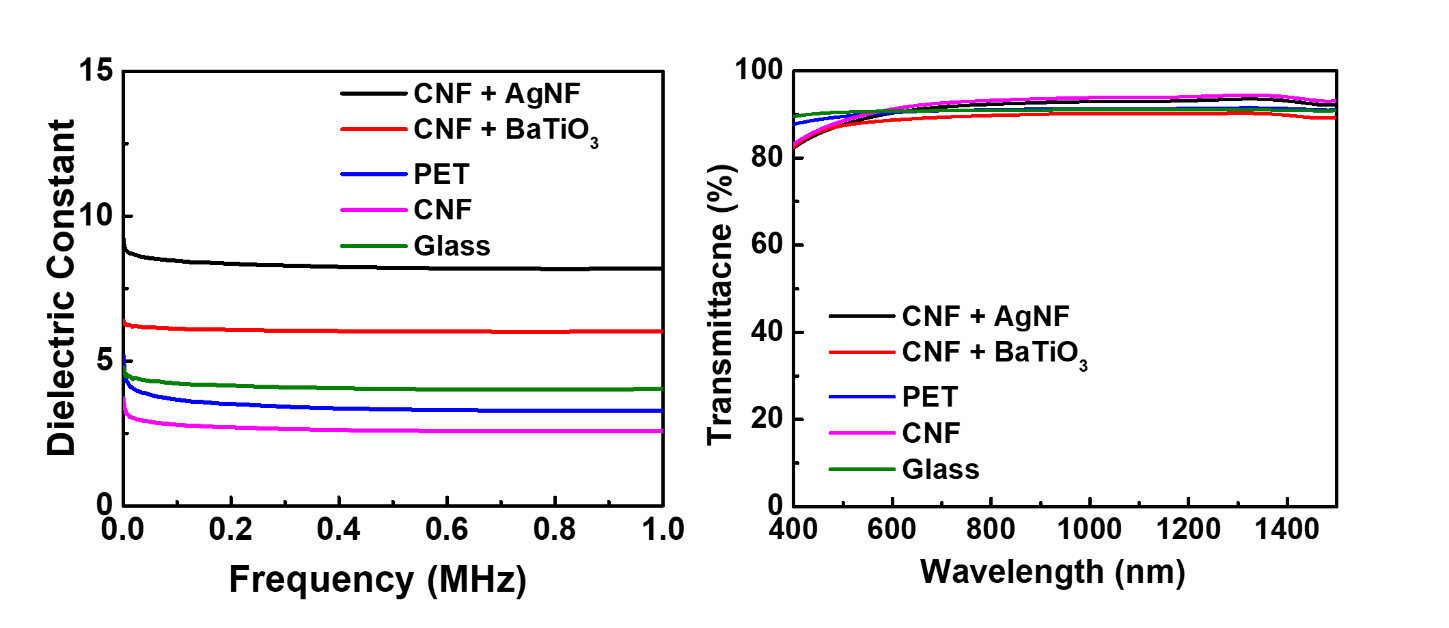


Supplementary Figure 12 │ Dielectric constant and transmittance of various cover layer film (Thickness: 100 μm)


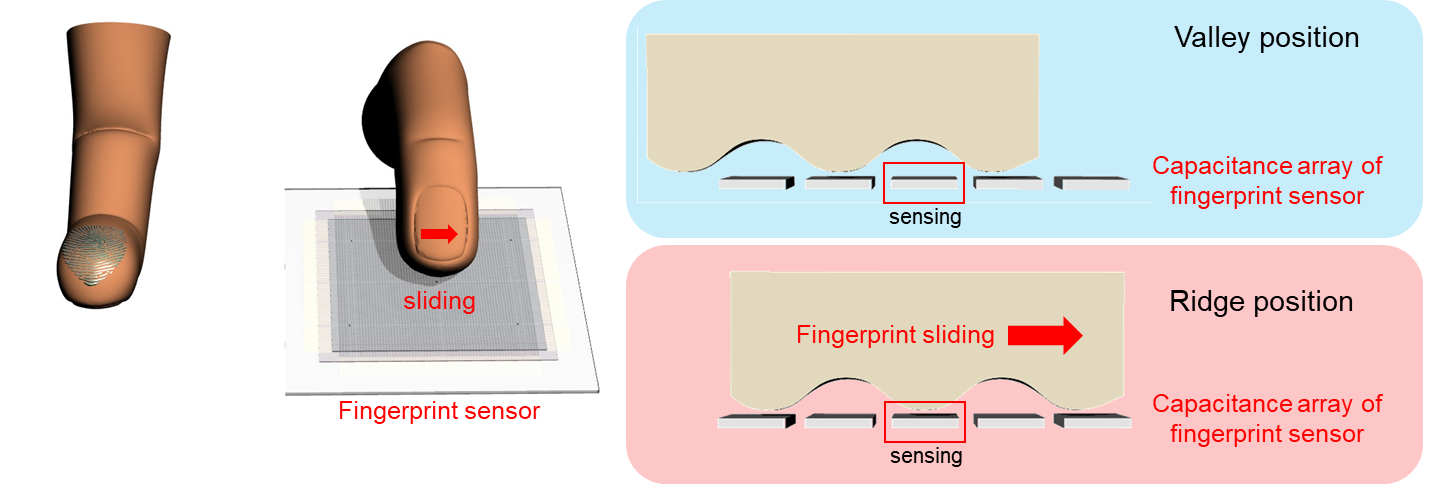


**Supplementary Figure 13 │ Schematic illustration of real-time fingerprint sensing for Figure 3e.**

**
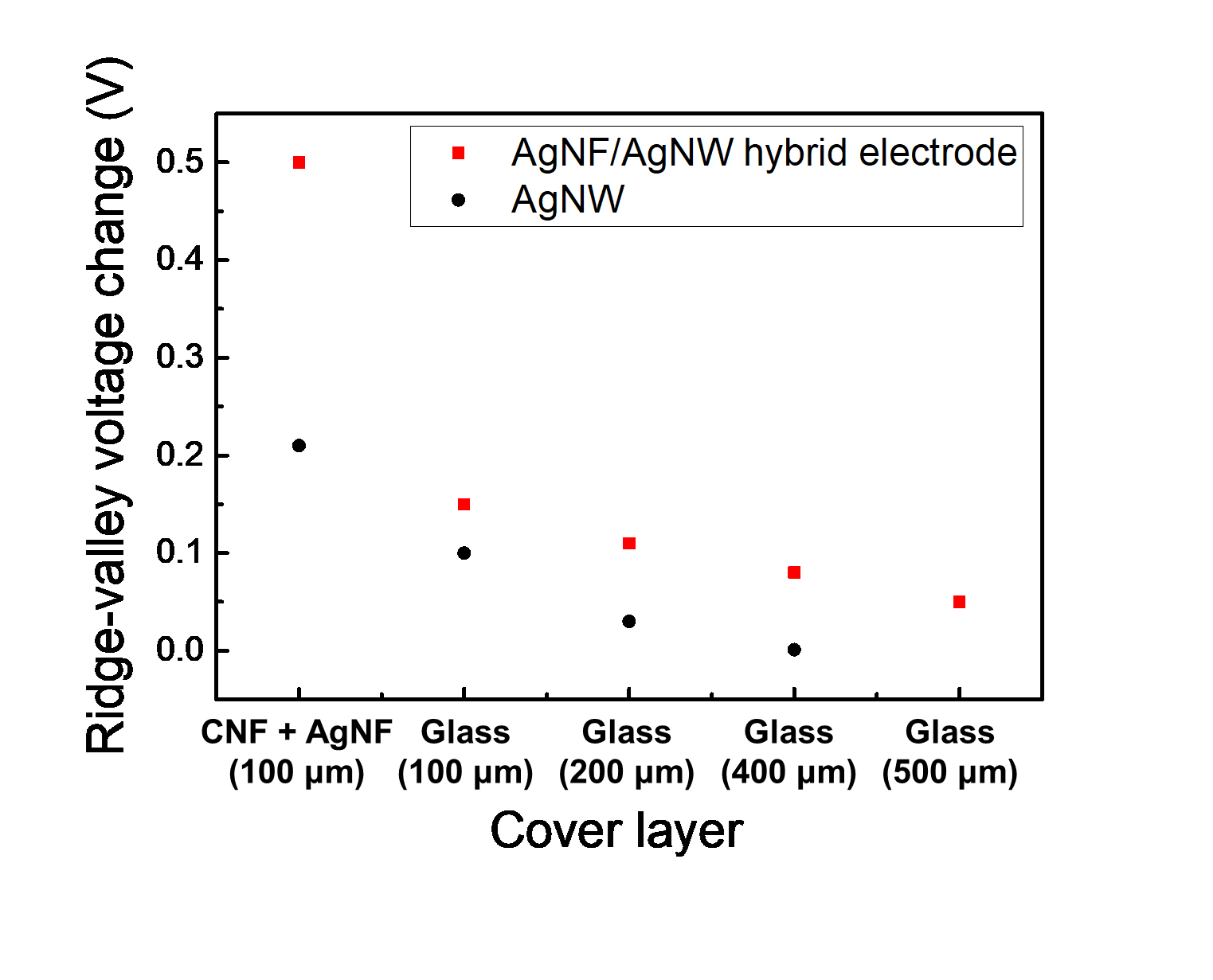
**

**Supplementary Figure 14 │ Comparison in output voltages of the fingerprint sensor fabricated using two different electrodes.** (i) AgNWs alone or (ii) the AgNF-AgNW hybrid. Here a glass (with diverse thicknesses) or the CNF+AgNF hybrid film (thickness: 100 μm) were used as the cover layer. The ridge-valley voltage change of the sensor using AgNWs with the 500 μm-thick glass cover was too small to be detected.


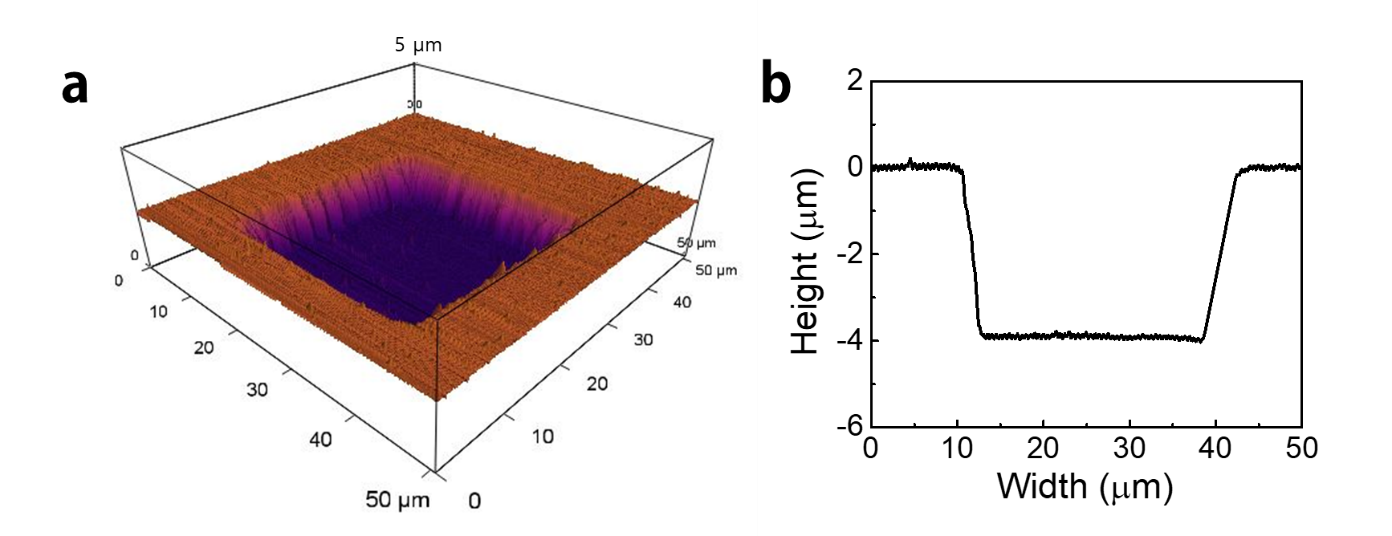


**Supplementary Figure 15 │ Air-gap dielectric layer. a,** AFM image of air dielectric. **b,** Cross-section profile of air dielectric.
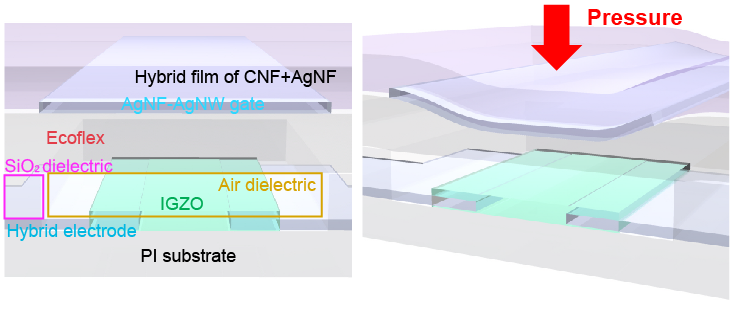


Supplementary Figure 16 │ Schematic illustration of pressure sensor and sensing mechanism.

Supplementary Figure 17 │ Patterned AgNF-AgNW hybrid electrode. SEM image of S/D electrodes of pressure-sensitive FETs between fingerprint sensor electrodes. Scale bar, 50 μm.


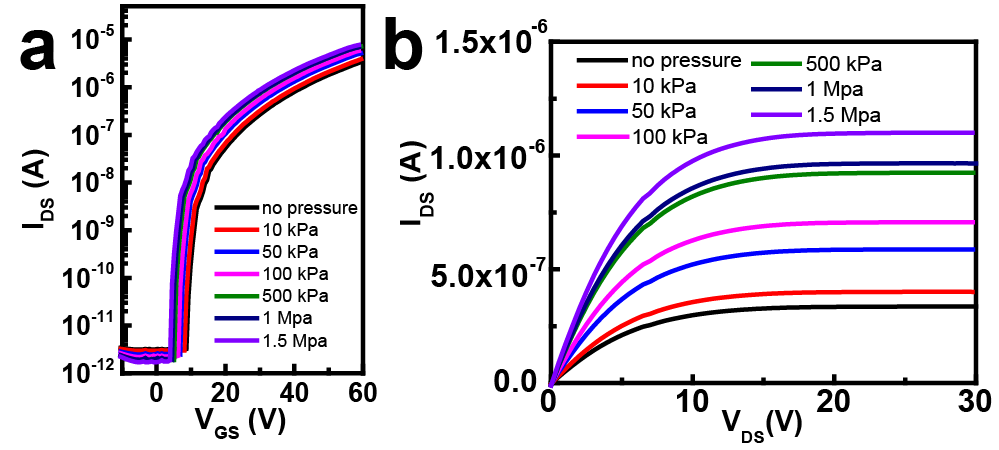


Supplementary Figure 18 │ Electrical properties of pressure sensitive transistor. a, Transfer characteristic of pressure sensor under different amounts of applied pressures from 0 kPa to 1.5 MPa (VDS = 10 V). b, Output characteristic of pressure sensor under different amounts of applied pressures from 0 kPa to 1.5 MPa (VD = 10 V, VG = 30 V).

**Supplementary Figure 19 │ Mechanical properties of Ecoflex.** Comparison between electrical responses of the fabricated pressure sensor and true stress-strain curve for an Ecoflex film compression test.


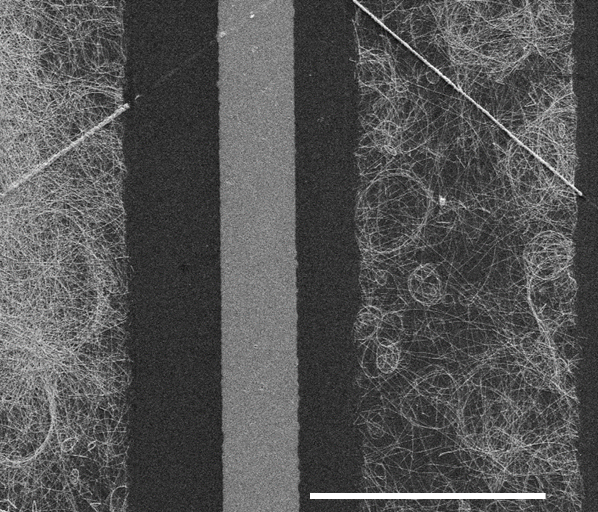


**Supplementary Figure 20 │ Patterened PEDOT:PSS line for temperature sensor.** SEM image of temperature sensor line between fingerprint sensor electrodes. Scale bar, 50 μm.


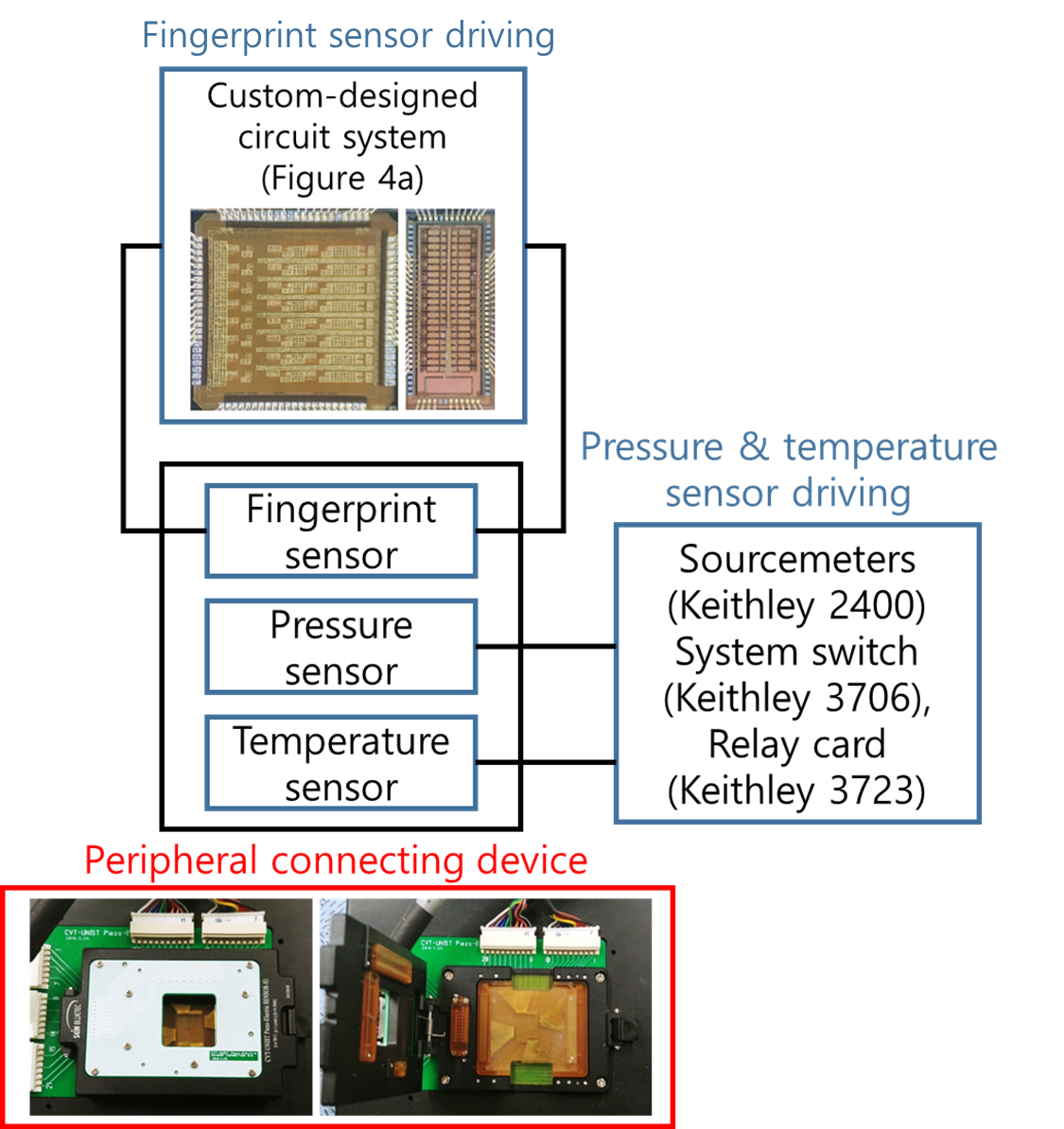


**Supplementary Figure 21 │ Circuit diagram of simultaneous detection system.**


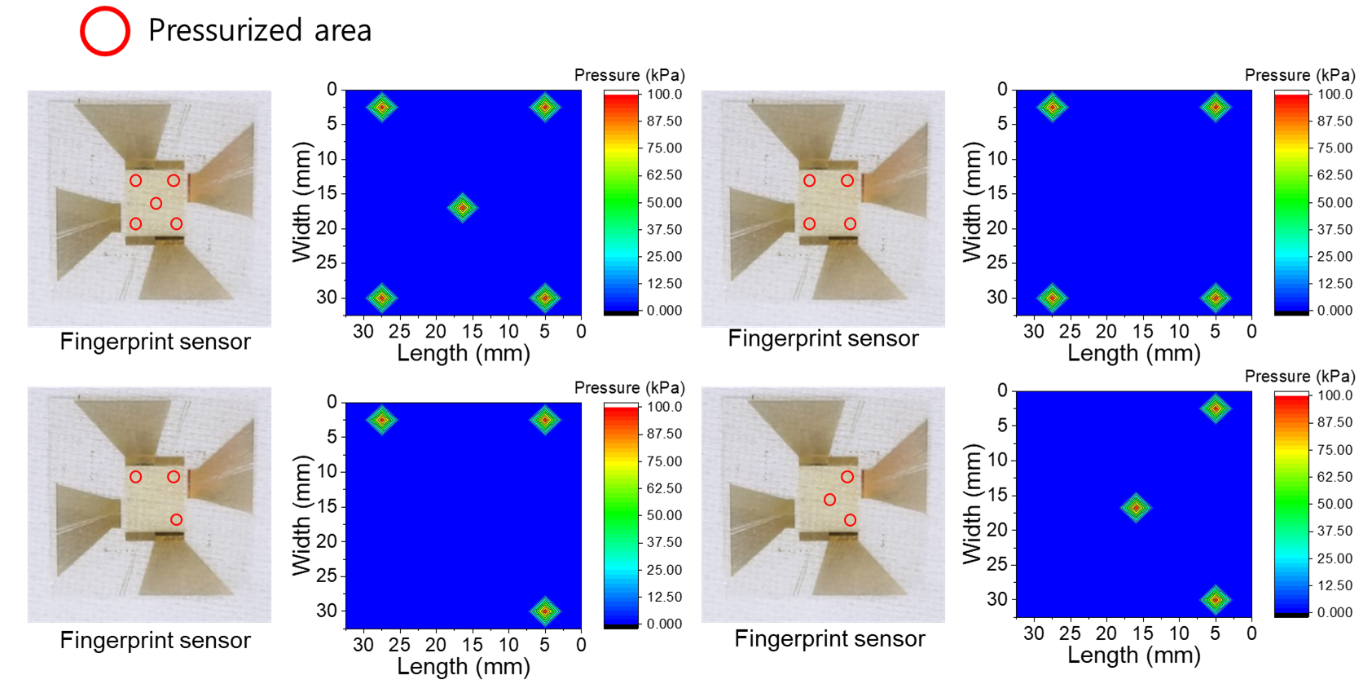


**Supplementary Figure 22 │ 2D mapping of tactile pressure using the pressure sensor array.**


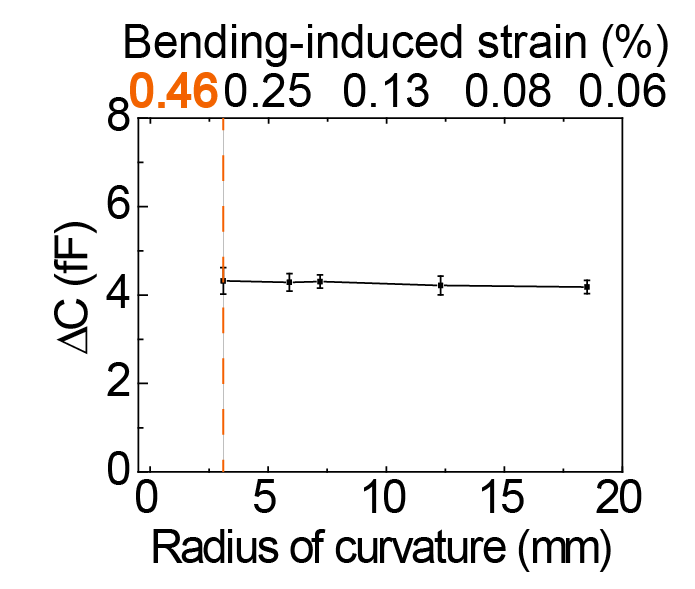


Supplementary Figure 23 │ Capacitance change between ridge and valley of fingerprint under various bending radii. The values of error bar of the capacitance are standard deviation. Same calculation method in supplementary method was used.

Supplementary Tables

| **Electrode** | **Sheet resistance (Ω/sq)**  **(Before patterning)** | **Sheet resistance (Ω/sq)**  **(After patterning)** | **Resistance of single electrode**  **(65** $\boldsymbol{\times}$ **11000 μm)** |
| --- | --- | --- | --- |
| AgNF | 5.1 $\pm$ 0.28 | **Infinite (undetectable)** | **Infinite (undetectable)** |
| AgNW | 11.3 $\pm$ 0.25 | 18.5 $\pm$ 1.25 | 3135.1 $\pm$ 123.52 |
| AgNF-AgNW | 1.03 $\pm$ 0.08 | 4.5 $\pm$ 0.65 | 765 $\pm$ 23.32 |

Supplementary Table 1 │ Comparison in sheet resistance and resistance of three different electrodes. (i) AgNFs alone, (ii) AgNWs alone, or (iii) AgNF-AgNW hybrid before and after patterning. Here the pattern dimension is 65 × 11000 μm, which is same as the fingerprint sensor electrode. The values of error bar of the resistance are standard deviation. Calculation was followed “error bar calculation” method in supplementary method.

|  | **Sheet resistance (Ω/sq)** | **Area fraction** |
| --- | --- | --- |
| Initial | 1.02 $\pm$ 0.07 | 0.055$\pm$ 0.003 |
| DI water | 1.03 $\pm$ 0.08 | 0.056 $\pm$ 0.003 |
| Acetone | 1.05 $\pm$ 0.07 | 0.055 $\pm$ 0.002 |
| IPA | 1.01 $\pm$ 0.07 | 0.057 $\pm$ 0.004 |
| Photoresist developer | 1.09 $\pm$ 0.1 | 0.054 $\pm$ 0.003 |

Supplementary Table 2 │ Electrical property of the AgNF-AgNW hybrid electrode after the immersion test. Changes in the sheet resistances and area fractions of the AgNW-AgNW hybrid networks after the immersion test. The values of error bar of the resistance are standard deviation. Calculation was followed “error bar calculation” method in supplementary method.
